# AI-Powered Noninvasive Electrocardiographic Imaging Using the Priori-to-Attention Network (P2AN) for Wearable Health Monitoring

**DOI:** 10.3390/s25061810

**Published:** 2025-03-14

**Authors:** Shijie He, Hanrui Dong, Xianbin Zhang, Richard Millham, Lin Xu, Wanqing Wu

**Affiliations:** 1 School of Biomedical Engineering, Sun Yat-sen University, Shenzhen 518107, China; heshj26@mail2.sysu.edu.cn (S.H.); donghr5@mail2.sysu.edu.cn (H.D.); zhangxb55@mail2.sysu.edu.cn (X.Z.); 2 Department of Information Technology, Durban University of Technology, Durban 4001, South Africa; richardm1@dut.ac.za; 3 General Hospital of the Southern Theatre Command, Guangzhou 510010, China; 4 The First School of Clinical Medicine, Southern Medical University, Guangzhou 510515, China

**Keywords:** health monitoring, cardiovascular disease, electrocardiographic imaging, cardiac electrophysiology, neural network

## Abstract

The rapid development of smart wearable devices has significantly advanced noninvasive, continuous health monitoring, enabling real-time collection of vital biosignals. Electrocardiographic imaging (ECGI), a noninvasive technique that reconstructs transmembrane potential (TMP) from body surface potential, has emerged as a promising method for reflecting cardiac electrical activity. However, the ECG inverse problem’s inherent instability has hindered its practical application. To address this, we introduce a novel Priori-to-Attention Network (P2AN) that enhances the stability of ECGI solutions. By leveraging the one-dimensional nature of electrical signals and the body’s electrical propagation properties, P2AN uses small-scale convolutions for attention computation, integrating a priori physiological knowledge via cross-attention mechanisms. This approach eliminates the need for clinical TMP measurements and improves solution accuracy through normalization constraints. We evaluate the method’s effectiveness in diagnosing myocardial ischemia and ventricular hypertrophy, demonstrating significant improvements in TMP reconstruction and lesion localization. Moreover, P2AN exhibits high robustness in noisy environments, making it highly suitable for integration with wearable electrocardiographic clothing. By improving spatiotemporal accuracy and noise resilience, P2AN offers a promising solution for noninvasive, real-time cardiovascular monitoring using AI-powered wearable devices.

## 1. Introduction

Cardiovascular disease remains one of the leading causes of death worldwide, as identified by the World Health Organization [1]. Early prevention and screening for risk factors such as myocardial ischemia (MI) [2,3] and ventricular hypertrophy (VH) [4] are critical to reduce morbidity and mortality. Electrocardiography (ECG) is a widely used tool for assessing cardiac health [5,6], and with the rise of smart wearable devices, continuous, real-time monitoring of ECG signals has become more accessible and convenient [7,8]. These wearable devices, embedded with high-resolution ECG sensors, offer the potential for noninvasive, continuous health monitoring, which is essential for early detection of cardiovascular diseases [9,10].

However, conventional 12-lead ECG systems have certain spatial resolution limitations in providing information on cardiac electrical activity [11,12]; electrocardiographic imaging (ECGI) enables 3D dynamic reconstruction of cardiac electrical activity at higher spatial resolution through inverse mapping of high-density body surface potentials [12,13,14]. This advancement offers a microscopic perspective for the diagnosis and localization of cardiac diseases as well as the study of cardiac electrical activity [12,15]. The integration of the algorithms with high-density BSP acquisition systems facilitates noninvasive, continuous, high-precision screening and localization of cardiac diseases, while revealing changes in cardiac function that are difficult to monitor with conventional 12-lead ECG. Additionally, this method avoids the need for costly imaging methods and invasive cardiac electrophysiological examinations. In this context, ECGI has gained attention as a noninvasive technique to provide detailed 3D maps of the heart’s electrical activity, known as transmembrane potential (TMP) [16,17,18]. And a large number of studies have demonstrated and supported the clinical utility of ECGI in more accurately understanding and localizing the underlying mechanisms of MI [19] and VH [20].

Recently, deep learning methods have showcased great efficacy in mitigating computational model errors in ECGI [12,18,21,22]. This can be attributed to the proficiency of deep learning models in addressing nonlinear complexities, such as intricate relationships within TMP distributions, along with their ability to effectively represent data across various levels of abstraction, thus accurately capturing salient data features [21,23]. However, it is essential to acknowledge that while deep learning methods excel in handling intricate data relationships, they may inadvertently overlook relevant physiological constraints, such as generating solutions beyond the electrophysiological range [12,13,23,24]. In certain instances, this oversight could yield outputs lacking meaningful interpretation and causing misunderstandings [23]. Therefore, it is crucial to emphasize the importance of balancing physiological and physical modeling constraints.

In this paper, we propose a method called Prior-to-Attention Network (P2AN) for realizing reconstruction from BSP to TMP. The method enhances the accuracy and physiological plausibility of the reconstruction by incorporating a priori physiological knowledge into the network structure based on the attention mechanism. Given the disparate attenuation properties exhibited by electrical activities in disparate regions of the heart when propagating to the body surface, the core structure of P2AN, a feature decoding module based on the attention mechanism, was designed. The objective of this module is to model the differential contribution of BSP from disparate locations to specific cardiac regions, thereby more accurately reflecting the propagation characteristics observed in cardiac electrophysiology. Furthermore, the standard TMP was incorporated into the model structure and objective function design as a priori knowledge. In the Decoder of P2AN, the encoded standard TMP is fused with the embedding BSP through a cross-attention mechanism, thereby ensuring that the information from the standard TMP can be used to guide the reconstruction process in each decoding step. This design effectively incorporates a priori physiological knowledge into the network, thereby providing a more accurate reference for reconstruction. In the construction of the objective function, in addition to including the forward propagation equation, we specifically introduce a mean square error optimization term between the reconstructed TMP and the standard TMP to constrain the morphological characteristics of the output signal. This constraint effectively limits the solution to the physiological space and ensures that the generated TMP is physiologically sound.

Subsequently, the results were reconstructed using an existing geometric model of the cardiac torso, enabling the visualization of the cardiac electrical activity. The extraction and analysis of the TMP features provided noninvasive access to comprehensive cardiac electrical information, offering a promising avenue for cost-effective diagnosis through visualization. This method allows for the precise screening and localization of adverse cardiac events, thereby improving the efficiency and accuracy of cardiac diagnosis.

Finally, in order to validate the accuracy and authenticity of the reconstructed TMP, a comparative analysis was performed between the proposed P2AN method and two benchmark methods documented in the literature (i.e., the ISTA [25,26,27] and FFNN [13,28] methods). The reconstruction performance of these methods was subjected to critical evaluation in different tasks, with two common types of heart disease (MI and VH) taken as examples, using relative errors (REs), correlation coefficients (CCs) and localization errors (LEs) for quantitative evaluation. The results demonstrate that our method exhibits superior accuracy and disease localization capabilities compared to the other two methods, and demonstrates high robustness under different noise levels.

The organization of this paper is as follows: Section 2 reviews the basic concepts and principles of ECGI and presents related works. Section 3 provides a detailed description of our work, including the details of the network, dataset description, and evaluation metrics. Section 4 demonstrates the performance of our method compared to other methods on the MI task, the VH task, and performance against noise. Section 5 discusses the strengths, limitations and the outlook for future work. Section 6 concludes the study.

## 2. Related Works

### 2.1. Electrocardiographic Imaging (ECGI)

ECGI is a method of solving inverse problems to reconstruct cardiac surface source signals from BSP at a given distance. To solve the inverse problem of ECG, which means to compute TMP from BSP, a forward problem model is first required. The forward problem of ECG can be summarized as the following linear equation:

(1) Ω=HU

where $U\in R^{M\times L}$ and $\Omega \in R^{N\times L}$ are TMP and BSP, respectively; *M*, *N*, and *L* are the number of cardiac mesh nodes, the number of body surface leads and the length of sampling time, respectively; and $H\in R^{N\times M}$ is the transfer factor obtained by the forward problem, which represents the forward mapping relationship from TMP to BSP. It is calculated by using boundary element method (BEM) [29] or finite element method (FEM) [30] utilizing the heart and torso geometry model. Relatively, the inverse problem of ECG estimates *U* from Ω. According to the Helmholtz principle [11], the inverse problem of ECG is ill posed. Although a solution for cardiac sources exists, it is not unique, nor does the solution depend continuously on the given data. Therefore, this underdeterminedness results in multiple solutions being possible. Additionally, due to the strong attenuation of the high-spatial-frequency components mapped onto the body surface, a small signal perturbation on the body could cause a nonlinear effect on the output because of signal amplification in the reconstruction process, thereby adversely affecting computation [12,29]. Consequently, in order to solve the inverse problem, a regularization term is incorporated based on a priori knowledge with the objective of constraining the solution of the equation to a reasonable margin of error [12,31].

### 2.2. Neural Network Methods

With the development of artificial intelligence, neural network-based ECGI methods have garnered significant attention and recognition, owing to their remarkable data learning and fitting capabilities [4,30].

A study by Joe Horvath et al. [28] demonstrated the prediction of cardiac surface potential utilizing a feed-forward neural network (FFNN) based on measurements obtained from the BSP. FFNN, as a fundamental neural network-based method, has found extensive application since the inception of the backpropagation learning algorithm. Jianxin Xie et al. [13] further built on the above framework by encoding and incorporating the fundamental physical laws of cardiac electrodynamics into the training process of the model, which demonstrated superior performance in several evaluation metrics.

Liansheng Wang et al. [25] introduced an ISTA-Net model for addressing the ECG inverse problem, leveraging its strong performance in image super-resolution within the ECG inverse problem domain. The two subproblems of ISTA algorithm [32] are solved alternatively to converge and obtain the ultimate solution:

(2) $$r^{\left(k\right)}=u^{\left(k-1\right)}-\rho \Phi^{⊤}\left(\Phi u^{\left(k-1\right)}-\omega \right)$$

and

(3) $$u^{\left(k\right)}=\arg\min_{u}\frac{1}{2}∥u-r^{\left(k\right)}{∥}_{2}^{2}+\lambda {∥\Psi u∥}_{1}$$

where *k* is the number of current iterations. Gradient descent is achieved by using (2), where Φ is the transfer matrix, $u^{\left(k-1\right)}$ is the result of the previous iteration, and ρ is the descent step. $u^{\left(k\right)}$ is reconstructed from $r^{\left(k\right)}$ by using (3), and Ψx is the regularization term, which is replaced in the network by F(u)=BReLU(Au), in which *A* and *B* are the two convolution operators. Subsequently, numerous researchers integrated novel modules into the ISTA method to enhance reconstruction outcomes, taking into account the data intrinsic characteristics and physiological properties. Lide Mu et al. [26] used graph convolution to enhance the spatial representation of the data on the basis of the ISTA method, while Shuting Xie et al. [27] added a non-local feature extraction module.

A review of related studies reveals that data-driven neural network-based models demonstrate robust feature learning capabilities and are capable of adaptively fitting data distributions. Nevertheless, there is still a paucity of targeted efforts to incorporate a priori physiological knowledge into the model learning process. The complexity of physiological mechanisms makes it challenging to identify relevant parameters [13]. Inspired by these works, we not only focus on the optimisation of traditional models but also revisit the spatiotemporal dynamics of BSP and TMP and integrate this a priori information with deep learning neural networks.

## 3. Methods

### 3.1. Data Collection

In this study, the Windows Release Version 3.0.1 of the ECGSIM software (December 2019) was used to obtain [BSP, TMP] data pairs, which is based on the principle of the ECG forward problem [33]. This software features low computational complexity (<1 s per case) and can run efficiently on any standard computer. And it provides the user with a dynamic interface to modify local source potential at the ventricular surface, along with the depolarization and repolarization time. Therefore, this simulation permits emulation of diverse adverse cardiac events by modulating local TMP and computing corresponding BSP. In this case, precise TMP-BSP data pairs for various simulated cardiac scenarios can be derived. Since the current version does not support batch processing, the acquisition of simulated data was performed manually on a case-by-case basis, ensuring the accuracy of the generated data. The representation of myocardial electrical activity within the ECGSIM calculation process is achieved through the equivalent double layer (EDL) model [34]. Additionally, the transfer factors governing the electrical propagation from TMP to BSP are meticulously computed upon authentic heart and torso geometries and the corresponding interfaces.

Our study focused on two typical cardiac adverse events: myocardial ischemia (MI) and ventricular hypertrophy (VH). MI manifests as a reduction in the action potential amplitude within the ischemic region, and the severity of this condition can be simulated by selecting a specific node on the heart surface and adjusting the amplitude of its action potential accordingly [35]. For our investigation, we simulated three varying degrees of MI by reducing the action potential by 25%, 50%, and 80%. Correspondingly, VH is characterized by an augmented thickness of the ventricular wall, leading to a prolonged depolarization [36]. Different degrees of VH were simulated in this study by selecting specific nodes on the heart surface and deliberately delaying their depolarization time. We emulated three degrees of VH by introducing delays of 25 ms, 40 ms, and 50 ms, respectively.

To validate the efficacy of the trained model in reconstructing TMP, we have set up four different types of datasets. Datasets 1 and 2 are used to characterize MI-type lesions and VH-type lesions, respectively. Datasets 3 and 4 add gradient signal-to-noise ratio (SNR) noise on the basis of Datasets 1 and 2. The datasets are introduced as follows:

- Dataset 1: 20 groups which were randomly selected from the 128 nodes in the epicardium, and the amplitude was arbitrarily adjusted to drop, ranging from 25% to 80%.
- Dataset 2: 20 groups which were randomly selected from the 128 nodes in the epicardium, and the depolarization time was arbitrarily delayed, ranging from 25 ms to 50 ms.
- Dataset 3: Add Gaussian noise with SNRs of 30 dB, 25 dB, 20 dB, and 15 dB, respectively, based on Dataset 1.
- Dataset 4: Add Gaussian noise with SNRs of 30 dB, 25 dB, 20 dB, and 15 dB, respectively, based on Dataset 2.

Dataset 1 and Dataset 2 were used to validate the reconstruction accuracy of the model as well as the recognition accuracy of MI and VH, respectively, while Dataset 3 and Dataset 4 test the model’s ability to combat noise.

### 3.2. Prior-to-Attention Network

**Figure 1:**
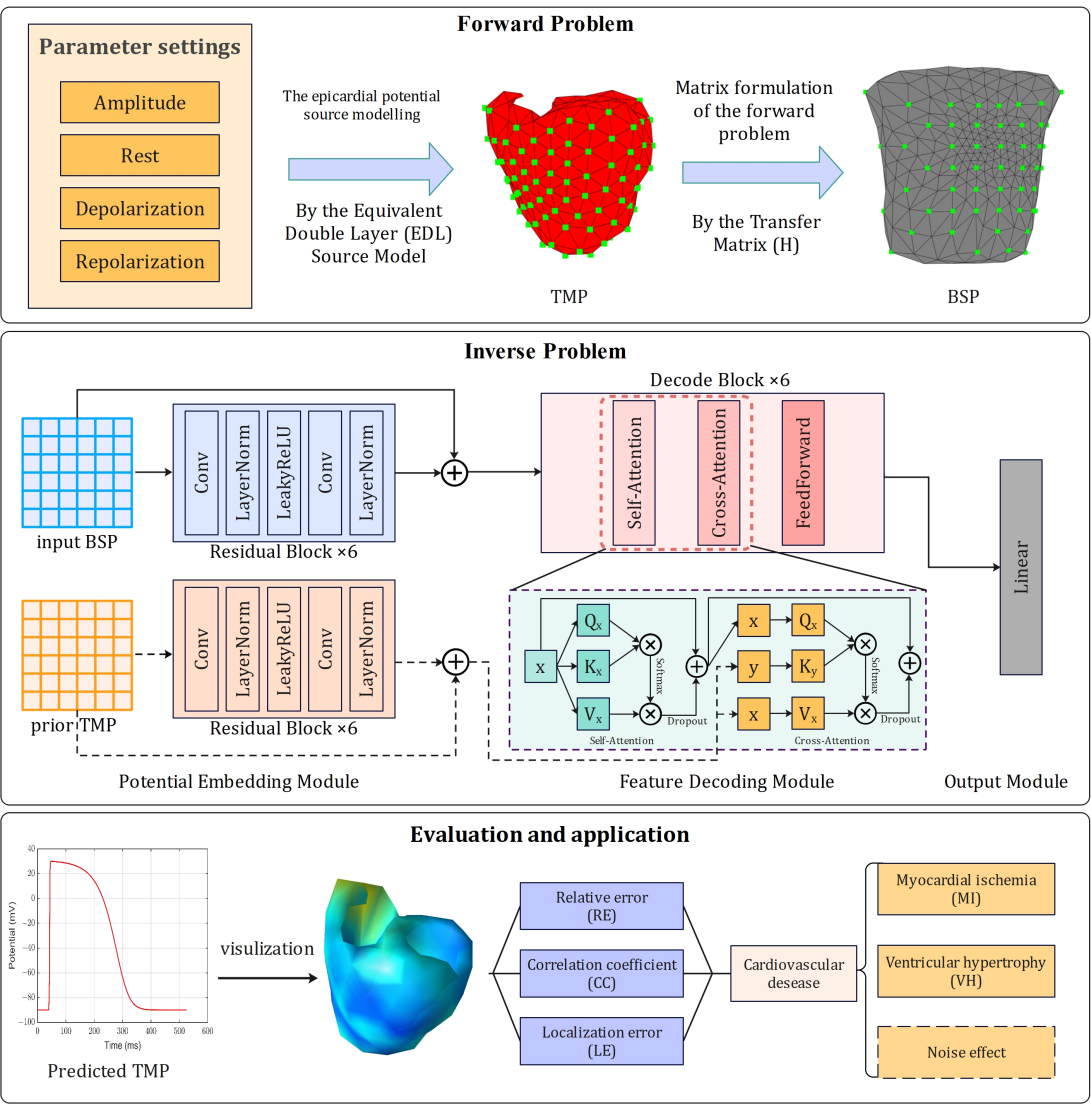
Overview of our research, including forward problem simulation, inverse problem modelling and evaluation and application of our method.

In this paper, the overall structure of our proposed P2AN can be divided into three main modules: potential embedding module, feature decoding module and output layer module (Figure 1). The specific design of each module is as follows.

The potential embedding module consists of stacked Residual Blocks designed to encode information from the input BSP signals and the a priori TMP signals. In each Residual Block, the input signals are processed by convolutional, normalization, activation, convolutional, and normalization layers for residual concatenation, which is calculated as follows:

(4) Em(x)=Norm(Conv(LeakyReLU(Norm(Conv(x)))))

In this case, the convolutional layer uses a one-dimensional convolution, a design that is more in line with the timing characteristics of electrical signals [37]. Due to the conductive properties of human tissues, the electrical activity of the heart is able to propagate to the body surface in a very short period of time (<50 ms) [38]. As a result, the numerical relationship between the BSP and TMP signals on the time axis is more aligned and focuses mainly on the information of the BSP signal within an adjacent short time window. Therefore, a small convolutional kernel of size 3 was used in the convolutional layer during the potential embedding process, and six Residual Blocks were stacked to bring the receptive field up to 50 ms in order to efficiently process the signals within the neighboring 50 ms time window. Meanwhile, considering the amplitude distribution characteristics of the electrical signal at different locations, the normalization layer uses LayerNorm.

The feature decoding module is borrowed from the Decoder module in Transformer [39,40], and its core mechanisms are self-attention and cross-attention. The computational process is as follows:

(5) $$ATTN\left(x,c\right)=Softmax\left(\frac{Q\left(x\right)K{\left(c\right)}^{T}}{d_{k}}\right)V\left(c\right)$$

where *x* and *c* are the embedding vectors. The first sublayer of the Decoder block performs the self-attention computation, where *Q*, *K*, and *V* are derived from the output of the previous layer, i.e., c=x; the second sublayer performs the cross-attention computation, where *Q* is derived from the the output of the previous sublayer, while *K* and *V* come from the embedding TMP vector. Subsequently, the final output of the block is obtained after processing in feed-forward layers. It can be expressed as

(6) $$FF\left(x\right)=\omega_{2}\cdot LeakyReLU\left(\omega_{1}\cdot x+b_{1}\right)+b_{2}$$

where ω and *b* are the weight and bias of linear layer. The feature decoding module consists of six Decoder blocks, where the cross-attention computation provides a priori knowledge for signal reconstruction. And the introduction of the Dropout layer enhances the generalization ability and robustness of the model.

The output layer module consists of a linear Layer for generating the final output signal.

In particular, it should be noted that the embedding BSP and TMP vectors do not need to be position-encoded. Because the numbering of the leads or positions of the BSP and TMP signals is only for the convenience of naming and arranging, the positions of the leads do not have a sequential order like the text data. For example, the order of either lead 1 or lead 2 is not important for the signal reconstruction process, and thus the introduction of position coding would rather disrupt the distributional properties of the leads.

In model training, the design of the objective function follows the goal of minimizing the error between the reconstructed TMP signal, i.e., the error between the theoretical BSP signal obtained by forward computation and the real BSP signal. It can be expressed mathematically as

(7) $$Loss_{forward}=||H\hat{u}-x{\left|\right|}_{2}$$

where H is the transfer matrix from the forward problem, $\hat{u}$ is the predicted TMP, and *x* is the input BSP.

Given the ill-posed nature of the inverse problem, there are multiple solutions in the high-dimensional space that may align with the actual BSP signal after forward computation. In order to address this issue, additional constraints are introduced in order to normalize the solutions. In particular, the standard TMP signal is incorporated into the constraint equation, which restricts the output signal to be consistent with the standard signal in terms of morphological features. This ensures that the reconstructed signal can match the underlying process of changes in cardiac electrical activity. The constrained loss function is defined as follows:

(8) $$Loss_{constraint}=\left|\right|\hat{u}-u{\left|\right|}_{2}$$

where *u* is the standard TMP.

Therefore, the final total loss function is

(9) $$Loss_{total}=Loss_{forward}+\lambda \cdot Loss_{constraint}$$

where λ is the weight factor with a setting of 0.01.

The training process has been presented in Algorithm 1. The algorithms in this study were developed based on the Nijmegen_64 electrode configuration, enabling high-density ECG signal acquisition through 64 electrodes. This configuration can be applied via traditional wet electrode arrays or integrated with novel dry electrode ECG suits [41,42] and is also compatible with commercially available systems, such as Medtronic CardioInsight^TM^ (Manufactured by CardioInsight Technologies, Inc., in Cleveland, OH, USA), offering flexible clinical options.
**Algorithm 1** Training process of P2AN1:**Load** Transfer matrix *H* and priori standard TMP $c_{raw}$2:**Initialize** parameters θ of P2AN3:**repeat**4:   Sample $x_{raw}$ from Training Datasets5:   // Prepare the cycle variable6:   $x\leftarrow x_{raw}$7:   $c\leftarrow c_{raw}$8:   // The first module: potential embedding module9:   //number of Residual block $n_{res}$10:   **for** i=1 to $n_{res}$ **do**11:     Calculate embedding by Equation (4)12:     x=x+Em(x)13:     c=c+Em(c)14:   **end for**15:   // The second module: feature decoding module16:   // Stack embedding bsp *x* to fit the size of TMP17:   x=concentrate((x,x),axis=1)18:   // number of Attention block $n_{attn}$19:   **for** i=1 to $n_{attn}$ **do**20:     // Calculate self-attention and cross-attention layer by Equation (5)21:     x1=Norm(x+Dropout(ATTN(x,x)))22:     x2=Norm(x+Dropout(ATTN(x1,c)))23:     // Calculate feed forward layer by Equation (6)24:     x=Norm(x2+Dropout(FF(x2)))25:   **end for**26:   // The third module: output layer module27:   y=ω·x+b28:   // Calculate loss $l_{forward}$ by Equation (7) and $l_{constraint}$ by Equation (8) with weight factor λ29:   $l_{total}=l_{forward}\left(y,x_{raw}\right)+\lambda \cdot l_{constraint}\left(y,c_{raw}\right)$30:   // Update parameters with learning rate α31:   $\theta^{*}\leftarrow \alpha \cdot \nabla l_{total}$32:**until** converged

### 3.3. Evaluation Metrics

In this study, we compared the P2AN method with ISTA [25,26,27] and FFNN [13,28] methods, which are two widely accepted and cited ECGI studies, to assess their reconstruction accuracy in solving the inverse problem. The evaluation of reconstruction accuracy of these inverse problem methods involves computing RE, CC and LE. Among them, RE and CC quantitatively evaluate the reconstructed waveforms as follows:

(10) $$RE=\frac{1}{N}\sum_{j=1}^{N}\sqrt{\frac{{\left({\hat{u}}_{i,j}-u_{i,j}\right)}^{2}}{{\left(u_{i,j}\right)}^{2}}}$$

and

(11) $$CC=\frac{1}{N}\sum_{j=1}^{N}\frac{\left({\hat{u}}_{i,j}-{\bar{\hat{u}}}_{i}\right)\left(u_{i,j}-{\bar{u}}_{i}\right)}{\sqrt{{\left({\hat{u}}_{i,j}-{\bar{\hat{u}}}_{i}\right)}^{2}{\left(u_{i,j}-{\bar{u}}_{i}\right)}^{2}}}$$

in which $\bar{\hat{u}}$ and $\bar{u}$ represent the mean values of the reconstructed TMP and the simulated TMP, respectively. Finally, RE provides insights into the accuracy of overall TMP reconstruction, whereas CC primarily gauges the correlation between the trends in TMP waveforms and the simulated TMP waveforms.

Additionally, *LE* quantifies the euclidean distance between the centroid of the reconstructed TMP lesion area and the corresponding node set in the simulation, which is expressed mathematically as

(12) $$LE=\sqrt{{\left(x_{\hat{u}}-x_{u}\right)}^{2}+{\left(y_{\hat{u}}-y_{u}\right)}^{2}+{\left(z_{\hat{u}}-z_{u}\right)}^{2}}$$

in which *x*, *y*, and *z* are the 3D coordinates of the node, and $\hat{u}$ and *u* pertain to the reconstructed TMP derived from the reconstruction and the simulated TMP obtained from the simulation, respectively.

We performed comprehensive data analysis and 3D visualization using MATLAB R2022b. By employing the triangular mesh data of the heart and torso, we constructed a geometrical model of both structures. Potentials were then projected onto the model’s surface, utilizing a defined color scheme to facilitate the visualization of the electrical activity in 3D. Moreover, we prioritize the RE and LE metrics, focusing on the TMP reconstruction accuracy and sensitivity to adverse events. Accordingly, we conducted a thorough statistical analysis of the RE and LE metrics. Group differences were evaluated using the *t*-test, with significance set at *p* < 0.05. Additionally, median and quartile values in box charts are presented, and the analysis process was executed using R 4.3.1.

## 4. Results

The reconstruction performance of our proposed method was evaluated on the MI task and the VH task on the aforementioned datasets. The overall reconstruction accuracy was assessed using RE and CC, while the disease localization accuracy was evaluated using LE. Additionally, the robustness was tested by adding random noise. The results demonstrated that the proposed method can effectively improve the spatiotemporal accuracy and robustness, when compared to two other classical methods. The details of results are described in the following.

### 4.1. Myocardial Ischemia

We assesses the reconstruction and localization accuracy of the methods for MI using Dataset 1.

**Figure 2:**
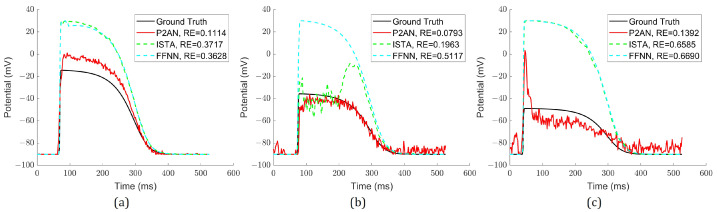
Simulated and reconstructed TMP waveforms through the P2AN, ISTA and FFNN methods with their RE at its core MI node. (**a**) With amplitude decrease of 33.3%; (**b**) with amplitude decrease of 45.8%; (**c**) with amplitude decrease of 66.7%.

Figure 2 illustrates the reconstructed waveforms through each method at the central MI node for varying degrees of MI, where the action potential amplitude decreased by 37.5%, 54.2%, and 66.7%. Evidently, the TMP reconstructed via the P2AN method consistently displayed a decrease in amplitude at the central node across all three MI degrees that was more similar to the simulated one. Furthermore, the RE for P2AN was notably smaller than that of the ISTA and FFNN methods, signifying superior temporal accuracy at the central node.

**Figure 3:**
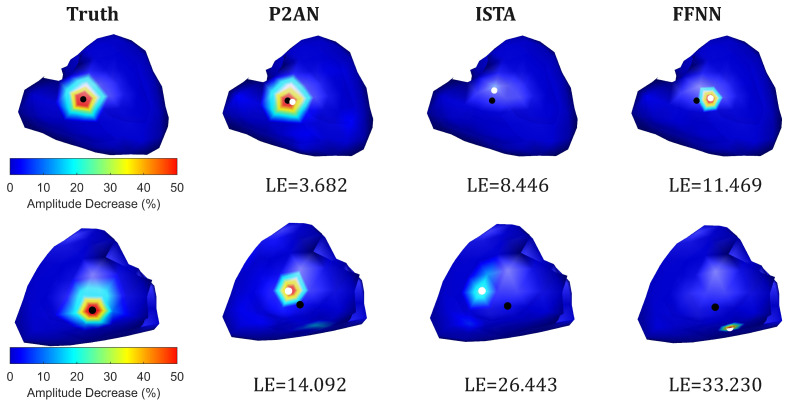
Visualization of the decrease in action potential amplitude (%) and comparison of the truth and reconstruction (through the P2AN, ISTA and FFNN methods) for its MI localization in the right (the first row) and left (the second row) ventricles.

For the one case depicted in Figure 3 (the first row), MI occurred in the right ventricle, as the figure shows in the anterior view. The LE values of the core lesion nodes obtained through the three methods and the set ground truth are 3.682 mm, 8.446 mm, and 11.469 mm, respectively. Similarly, for another case shown in Figure 3 (the second row) where the lesion occurred in the middle part of the left ventricle (posterior view), the LE values were 14.092 mm, 26.443 mm, and 33.230 mm, respectively. Overall, the TMP reconstructed using the P2AN method demonstrated superior spatiotemporal accuracy in waveform and localization.

**Figure 4:**
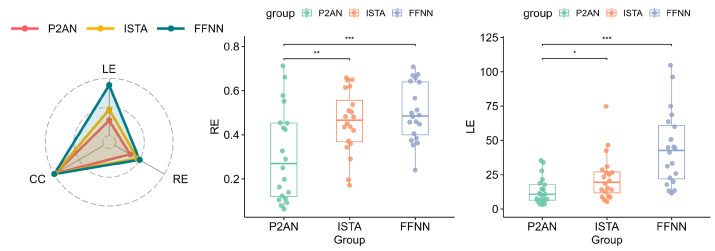
Visualization of evaluation metrics for reconstructed TMP in MI task through the P2AN, ISTA, FFNN methods (no significance, i.e., *p* > 0.05. * *p* < 0.05. ** *p* < 0.01. *** *p* < 0.001).

Figure 4 presents RE, LE and CC metrics for 20 groups of TMP reconstructed using P2AN, ISTA, and FFNN. As can be observed from the radar chart, the P2AN method demonstrates the lowest average values of RE and LE, despite the lack of a significant difference in average CC values among the three methods. Through a *t*-test, the reconstructed TMP by P2AN notably exhibits enhanced overall RE accuracy in comparison to both ISTA (*p* < 0.01) and FFNN (*p* < 0.001) methods. Also, the P2AN method significantly excels in lesion localization compared to ISTA (*p* < 0.05) and FFNN (*p* < 0.001) methods.

Table 1 provides a summarized view of the RE, CC, and LE across all 20 groups for the three methods. The CC indicates that all methods can effectively reconstruct TMP trends; however, the P2AN method outperforms in overall reconstruction accuracy and lesion localization.

### 4.2. Ventricular Hypertrophy

We assesses the reconstruction and localization accuracy of the methods for VH using Dataset 2.

**Figure 5:**
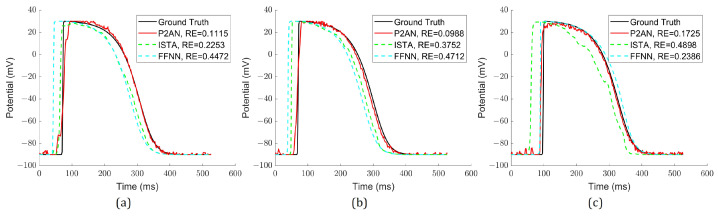
Simulated and reconstructed TMP through the P2AN, ISTA and FFNN methods with their RE at its core VH node. (**a**) With depolarization time delay of 30 ms; (**b**) with depolarization time delay of 35 ms; (**c**) with depolarization time delay of 45 ms.

Figure 5 displays the reconstructed waveforms of each method at its respective core node for varying degrees of VH, characterized by a delay in depolarization time (30 ms, 35 ms, and 40 ms). The TMP reconstructed by P2AN closely aligns with simulated waveforms at the core node across all three VH degrees, showcasing a smaller RE compared to TMP reconstructed by ISTA and FFNN. This emphasizes P2AN’s heightened temporal accuracy at the core node.

**Figure 6:**
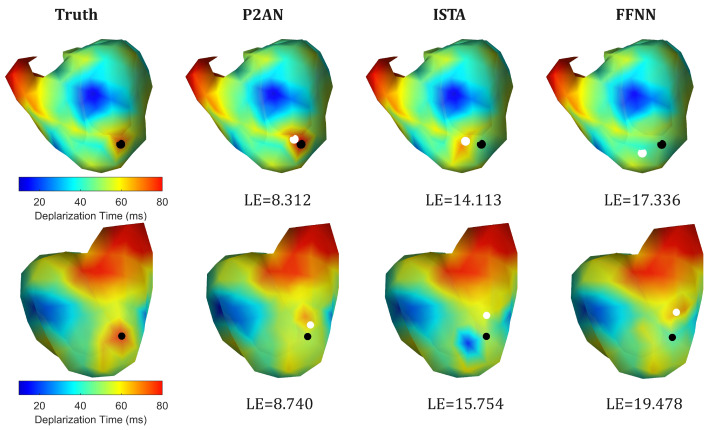
Visualization of the depolarization time (ms) in action potential and comparison of the truth and reconstruction (through the P2AN, ISTA and FFNN methods) for its VH localization in the left (the first row) and right (the second row) ventricles.

The first row of Figure 6 illustrates the 3D visualization of cardiac depolarization time in a case with VH in the left ventricle. For the three methods, LE values are 8.312 mm, 14.113 mm, and 17.336 mm, respectively. In the second row of Figure 6, LE values are 8.740 mm, 15.754 mm, and 19.478 mm, respectively. In both cases, the smaller LE values further emphasize the P2AN method’s heightened sensitivity to lesions.

**Figure 7:**
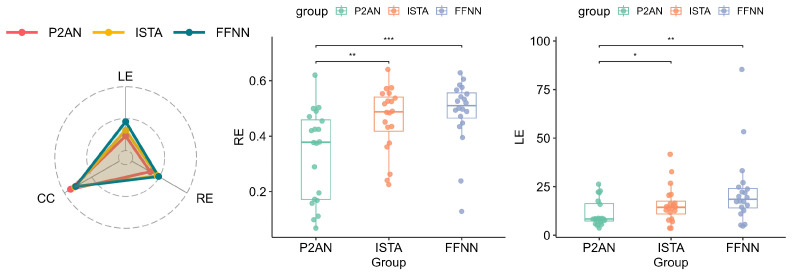
Visualization of evaluation metrics for reconstructed TMP in VH task through the P2AN, ISTA, and FFNN methods (no significance, i.e., *p* > 0.05. * *p* < 0.05. ** *p* < 0.01. *** *p* < 0.001).

Figure 7 presents 20 groups’ RE, LE and CC of reconstructed TMP using P2AN, ISTA, and FFNN methods. The radar chart demonstrates that the P2AN method yielded lower average values for both RE and LE, accompanied by higher average values for CC. This indicates a reduction in accuracy errors and localization errors and an enhanced trend fit. And the box chart shows that the TMP reconstructed via P2AN significantly outperforms ISTA and FFNN methods, displaying superior RE (*p* < 0.01, *p* < 0.001) and LE (*p* < 0.05, *p* < 0.01).

Table 2 summarizes the three metrics for all 20 groups across the three methods. In summary, the P2AN method not only better represents the TMP trend but also achieves higher precision in potentiometric values and greater sensitivity to the VH site.

### 4.3. Noise Effect

During the actual acquisition of physiological signals, inevitable disturbances introduce roughness and ineffective components, hindering meaningful analysis. Therefore, we assess the robustness of the three methods to varying noise levels (30 dB, 25 dB, 20 dB, 15 dB) using Dataset 3 and Dataset 4.

**Figure 8:**
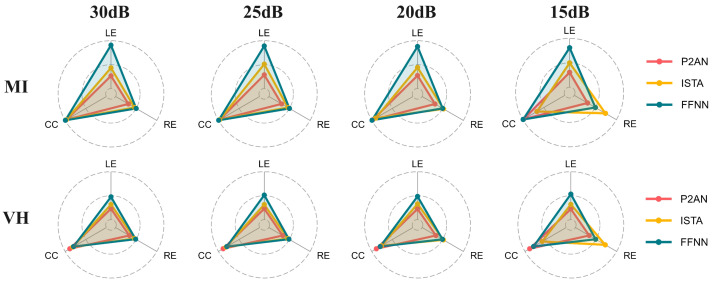
Visualization of evaluation metrics for reconstructed TMP in MI and VH task through the P2AN, ISTA, FFNN methods at signal-to-noise ratios of 30 dB, 25 dB, 20 dB and 15 dB.

Figure 8 depicts the visualized radargrams of the evaluation metrics for the various methods at different SNRs. In the MI task, the P2AN method demonstrates the lowest average RE and LE values, while the average CC values of the three methods are comparable. However, the metrics of the ISTA method exhibit considerable fluctuations at the 15 dB SNR level, indicating that ISTA becomes less effective in a strong noise background. In the VH task, the P2AN method demonstrated the most favourable results, exhibiting the lowest average RE and LE values and the highest CC value. Similarly, the presence of strong noise at 15 dB SNR has the greatest impact on the ISTA method.

**Figure 9:**
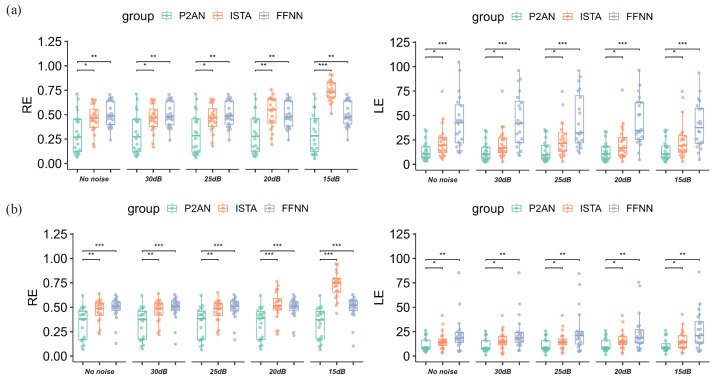
RE and LE values for reconstructed TMP in MI task through the P2AN, ISTA, and FFNN methods at signal-to-noise ratios of 30 dB, 25 dB, 20 dB and 15 dB. (**a**) MI task; (**b**) VH task (no significance i.e., *p* > 0.05. * *p* < 0.05. ** *p* < 0.01. *** *p* < 0.001).

The distributions of the P2AN method, as illustrated in Figure 9, demonstrate a consistent interval level across different SNRs, whereas the distributions of the ISTA method exhibit considerable variation. The performance of the P2AN method is significantly superior to that of the ISTA method (RE: *p* < 0.05 for MI and *p* < 0.01 for VH; LE: *p* < 0.05 for both MI and VH) and FFNN method (RE: *p* < 0.01 for MI and *p* < 0.001 for VH; LE: *p* < 0.001 for MI and *p* < 0.01 for VH). In addition, Cohen’s d was calculated for each pair. All calculated Cohen’s d values were greater than 0.8 in absolute value, indicating a significant effect size for the between-group differences, which ensured the statistical efficacy of the hypothesis test and resulted in high reliability and statistical significance of the *p*-values.

Table 3 and Table 4 provide a summary of the average and standard deviation of the evaluated metrics at different SNR levels for the MI and VH task, respectively. In general, both the P2AN method and the FFNN method demonstrate robust performance, whereas the ISTA method is less resilient to strong noise. However, the FFNN method exhibits inferior performance compared to the ISTA method at SNRs exceeding 15 dB. The P2AN method maintains robust performance while also demonstrating satisfactory reconstruction performance.

## 5. Discussion

In this study, we propose a P2AN approach centered around a priori knowledge and an attention mechanism, designed to improve the spatiotemporal accuracy of noninvasive ECGI reconstruction while enhancing robustness to random noise. The P2AN method is grounded in reliable forward problem theory, thus eliminating the need for an end-to-end deep learning paradigm. Furthermore, it does not rely on realistically captured TMP data during training, avoiding the high costs and invasiveness associated with TMP data collection.

In terms of model architecture, we exploit the one-dimensional temporal properties of electrical signals and employ one-dimensional convolution for feature extraction. An attention mechanism is incorporated to account for the fact that different body surface locations reflect varying degrees of electrical activity on the heart’s surface, thereby contributing different amounts of information to the inverse problem. To guide the reconstruction process, we introduce the standard TMP as a priori knowledge and encode it into the model using a cross-attention mechanism.

Additionally, we design two types of loss functions: the forward propagation loss and the constraint loss. The forward propagation loss facilitates training without real TMP data by incorporating the transfer matrix from the forward problem, while the constraint loss incorporates a priori standard TMP. This loss is included in the final objective function with a small weight factor to help constrain the morphological distribution of the output signal. Since multiple solutions may satisfy the forward propagation process in 3D space even when the forward propagation loss is minimized, a priori cross-attention is employed to allow the model to learn the TMP’s morphological distribution. This further restricts the spatial distribution of the output signals through an additional constraint.

A comparative analysis was therefore conducted between the proposed P2AN method and two established methods, namely the ISTA and FFNN methods, as documented in the literature. The reconstruction performance of these methods was subjected to critical evaluation in different tasks, and the performance of the three methods was assessed with two common types of cardiac diseases (MI and VH) as examples using three key metrics: RE, CC and LE. In general, all three methods were capable of reconstructing the acquired TMP-like signals and effectively representing the pathological characteristics inherent to the test data. It is noteworthy that the P2AN method exhibits a markedly lower RE and LE in comparison to the other two methods, which suggests enhanced numerical accuracy and precision in disease localization. With regard to the CC value, which reflects the trend of the reconstructed signals, all three methods demonstrated high performance, with the P2AN method exhibiting particular advantage in the VH task. Furthermore, the performance of the three methods was evaluated in the presence of noise-contaminated BSP data, using gradient SNR BSP data for testing. The results demonstrate that the P2AN method retains its superior accuracy and localization advantages over the remaining two methods, exhibiting high robustness under different noise levels.

Although the reliability of the P2AN method has been tested with various data types, it is important to recognize its limitations. Due to the difficulty in directly acquiring TMP in vivo, BSP mapping is used as an alternative for ECGI. We also acknowledge that ECGI has long been criticized for various challenges, including systematic errors, motion artifacts, and electrode misplacement. These issues have been persistent limitations in ECGI applications. However, at present, the best solutions to address these challenges can be achieved through data processing and analysis. Therefore, to simulate real-world conditions, we incorporated random Gaussian noise into the dataset, which helped us assess the algorithm’s robustness [43]. The results demonstrated that the algorithm maintains good stability and resilience under noisy conditions. Regarding the issue of electrode misplacement, using interpolation methods to correct minor electrode displacements can be an effective approach [44]. However, the resulting lack of information from misaligned leads remains unavoidable. Additionally, there is ongoing debate about the minimum electrode configuration [45,46], and we anticipate that future studies will further optimize electrode placement to enhance the accuracy and applicability of ECGI systems. Furthermore, while this study primarily highlights the exceptional performance of the P2AN method on the MI and VH tasks, it is important to emphasize that we are proposing a methodology rather than a disease-specific solution. In future research, pathological data such as atrial fibrillation, bundle branch block, and other conditions can be obtained from ECGSIM and seamlessly integrated into the training process using our proposed method, thereby enhancing the algorithm’s potential to adapt to diverse clinical scenarios. Therefore, future research will focus on enhancing the generalization ability by simulating a wider range of cardiac conditions and incorporating diverse heart–torso geometries. Despite the challenges of obtaining clinical TMP data, other investigative tools, such as CT and MRI imaging techniques [20,43], may serve as complementary methods for validating the findings.

With further refinement, it is poised to revolutionize noninvasive screening and diagnosis of adverse cardiac events, presenting a more information-rich and comprehensive screening method than conventional ECG examinations [47,48]. And with the increasing adoption of smart wearable devices for continuous health monitoring, integrating such deep learning techniques with wearable electrocardiographic clothing represents a significant advancement. These devices can offer real-time, noninvasive cardiac monitoring by continuously collecting high-resolution ECG data. This integration allows for precise, low-cost cardiac event screening and localization, bringing ECGI closer to practical, everyday use in clinical and home settings. Our results demonstrate that P2AN significantly outperforms existing methods in both accuracy and robustness, suggesting its potential for deployment in wearable ECG devices for enhanced cardiovascular health monitoring.

## 6. Conclusions

We proposed the P2AN method for noninvasively reconstructing cardiac electrical activity from BSP. By incorporating a priori knowledge and attention mechanisms, P2AN optimizes the ill-posed nature of the inverse problem, enhancing spatiotemporal accuracy and solution stability. This approach eliminates the need for clinical invasive measurements and enables the reconstruction of internal heart electrophysiology, offering a safer, lower-cost alternative to invasive techniques. Our method holds significant potential for integration with AI-powered wearable ECG devices, enabling continuous, real-time heart monitoring. It can detect subtle cardiac abnormalities that conventional ECG may miss, improving early detection of adverse events. As wearable ECG technology advances, P2AN can contribute to personalized cardiovascular care and enhance research into cardiac physiology.

## Figures and Tables

**Table 1:** Summary of the RE, CC and LE values with standard deviation in parentheses in MI task.

	P2AN	ISTA	FFNN
RE	0.310 (0.204)	0.459 (0.139)	0.510 (0.127)
CC	0.973 (0.048)	0.974 (0.055)	0.995 (0.012)
LE (mm)	13.439 (9.477)	22.920 (16.236)	44.142 (26.404)

**Table 2:** Summary of the RE, CC and LE values with standard deviation in parentheses in VH task.

	P2AN	ISTA	FFNN
RE	0.335 (0.159)	0.462 (0.113)	0.487 (0.117)
CC	0.887 (0.085)	0.815 (0.079)	0.796 (0.076)
LE (mm)	11.414 (6.742)	15.829 (9.113)	22.630 (17.859)

**Table 3:** Summary of the RE, CC and LE values with standard deviation in parentheses in MI task at different levels of noise.

		P2AN	ISTA	FFNN
30 dB	RE	0.312 (0.202)	0.460 (0.138)	0.500 (0.128)
	CC	0.973 (0.050)	0.973 (0.056)	0.995 (0.013)
	LE (mm)	13.244 (9.645)	21.605 (15.784)	45.339 (26.558)
25 dB	RE	0.310 (0.206)	0.462 (0.137)	0.500 (0.129)
	CC	0.974 (0.045)	0.973 (0.056)	0.994 (0.013)
	LE (mm)	14.308 (10.834)	25.534 (17.932)	44.514 (27.328)
20 dB	RE	0.313 (0.205)	0.519 (0.161)	0.496 (0.131)
	CC	0.979 (0.034)	0.904 (0.110)	0.994 (0.014)
	LE (mm)	13.594 (9.635)	22.089 (16.567)	43.942 (24.339)
15 dB	RE	0.316 (0.208)	0.740 (0.102)	0.502 (0.126)
	CC	0.984 (0.024)	0.665 (0.153)	0.992 (0.020)
	LE (mm)	15.166 (10.578)	24.888 (19.245)	40.429 (23.307)

**Table 4:** Summary of the RE, CC and LE values with standard deviation in parentheses in VH task at different levels of noise.

		P2AN	ISTA	FFNN
30 dB	RE	0.334 (0.159)	0.461 (0.114)	0.486 (0.118)
	CC	0.888 (0.085)	0.816 (0.079)	0.796 (0.076)
	LE (mm)	11.334 (6.858)	9.240 (15.764)	23.736 (18.062)
25 dB	RE	0.336 (0.160)	0.461 (0.112)	0.487 (0.111)
	CC	0.886 (0.085)	0.816 (0.079)	0.797 (0.075)
	LE (mm)	11.362 (6.865)	15.711 (8.971)	25.597 (21.224)
20 dB	RE	0.334 (0.158)	0.517 (0.147)	0.492 (0.104)
	CC	0.888 (0.084)	0.764 (0.119)	0.794 (0.072)
	LE (mm)	11.137 (6.659)	16.327 (9.322)	24.197 (18.939)
15 dB	RE	0.334 (0.159)	0.725 (0.132)	0.490 (0.121)
	CC	0.888 (0.085)	0.584 (0.138)	0.792 (0.077)
	LE (mm)	11.036 (6.709)	15.869 (9.714)	26.596 (19.670)

## Data Availability

The data presented in this study are available in “ECGSIM: an interactive tool for studying the genesis of QRST waveforms” at https://doi.org/10.1136/hrt.2003.014662, reference number [33]. These data were derived from the following resources available in the public domain: https://www.ecgsim.org/ (accessed on 30 May 2024).
